# Data-driven haemodynamic response function extraction using Fourier-wavelet regularised deconvolution

**DOI:** 10.1186/1471-2342-8-7

**Published:** 2008-04-10

**Authors:** Alle Meije Wink, Hans Hoogduin, Jos BTM Roerdink

**Affiliations:** 1 Robert Steiner MR Unit, Imaging Sciences Department Imperial College, and MRC Clinical Sciences Centre, Hammersmith Hospital, London, UK; 2 Institute for Behavioral and Cognitive Neurosciences and BCN Neuroimaging Center, Groningen, The Netherlands; 3 Institute for Mathematics and Computing Science, University of Groningen, The Netherlands

## Abstract

**Background:**

We present a simple, data-driven method to extract haemodynamic response functions (HRF) from functional magnetic resonance imaging (fMRI) time series, based on the Fourier-wavelet regularised deconvolution (*ForWaRD*) technique. HRF data are required for many fMRI applications, such as defining region-specific HRFs, effciently representing a general HRF, or comparing subject-specific HRFs.

**Results:**

*ForWaRD *is applied to fMRI time signals, after removing low-frequency trends by a wavelet-based method, and the output of *ForWaRD *is a time series of volumes, containing the HRF in each voxel. Compared to more complex methods, this extraction algorithm requires few assumptions (separability of signal and noise in the frequency and wavelet domains and the general linear model) and it is fast (HRF extraction from a single fMRI data set takes about the same time as spatial resampling). The extraction method is tested on simulated event-related activation signals, contaminated with noise from a time series of real MRI images. An application for HRF data is demonstrated in a simple event-related experiment: data are extracted from a region with significant effects of interest in a first time series. A continuous-time HRF is obtained by fitting a nonlinear function to the discrete HRF coeffcients, and is then used to analyse a later time series.

**Conclusion:**

With the parameters used in this paper, the extraction method presented here is very robust to changes in signal properties. Comparison of analyses with fitted HRFs and with a canonical HRF shows that a subject-specific, regional HRF significantly improves detection power. Sensitivity and specificity increase not only in the region from which the HRFs are extracted, but also in other regions of interest.

## 1 Background

In functional magnetic resonance imaging (fMRI), local brain activation temporally changes blood oxygenation, which induces a blood oxygenation level dependent (BOLD) contrast in MR images [1]. Given a model of the BOLD response to a stimulus pattern, statistics can be used to quantify the match between the predicted and measured signals in a voxel, and significant activation is assessed via hypothesis testing. Statistical parametric mapping (SPM) estimates parameters of the noise distribution in every voxel to determine a threshold for the computed statistic [2]. It uses the general linear model (GLM): the response to a stimulus pattern is modelled as the output of a linear, time invariant (LTI) system [3]. The response to one type of stimulus is modelled by convolving its temporal distribution with the response function of that type of stimulus. The total response is the sum of responses to all individual stimulus types. The stimulus pattern is known from the experimental setup. However, the haemodynamic response function (HRF), i.e., the temporal BOLD impulse response, is unknown. Essentially, it is a smooth curve that starts to rise two seconds after the stimulus, peaks after six seconds, and returns to baseline within 30 seconds.

The resolution of discrete fMRI time samples yields a coarse description of the HRF, which is a problem for designs with short and/or randomised stimuli. In slice-wise MRI acquisition, there is no optimal sampling for all slices, and slight differences in temporal onset between voxels in different slices must be accurately modelled to obtain good sensitivity. This requires a continuous-time HRF model. It is sometimes preferable to use a common HRF for many experiments and many regions. In many cases though, a common HRF cannot be assumed [4,5]. Two ways to solve this problem are (*i*) including a HRF model with more basis functions [5], and (*ii*) estimating the shape of the HRF in the statistical analysis [4,6]. A drawback of the first and simple approach is that it covers only limited variation in the HRF shape, while more advanced approaches suffer from high complexity and long computation times. For previous work on the variability of the BOLD response between brain areas and even across repeated measurements of the same subject, see Neumann et al. [7].

Extracting a HRF from fMRI data requires assumptions about its shape, and is computationally expensive. A simple method described in the literature is selective averaging with a long interstimulus interval (ISI), assuming non-overlapping responses [8,9]. Trials *with *overlapping responses are also averaged, ignoring the fact that the overlaps introduce errors [3,10,11]. Other studies use a functional description of the HRF, whose parameters are determined by curve fitting (examples in [12-14]), one of which [12] uses frequency-domain deconvolution. Another technique based on the GLM expands the HRF into a set of basis functions [15,16].

Ciuciu et al. [4] use a Bayesian method to extract the HRF. The method can simultaneously extract multiple HRFs from multiple experiments. Woolrich et al. [6] use a fully Bayesian approach to fMRI modelling, including the HRF. Both these approaches use certain prior assumptions for the shape of the HRF: in the first case, causality, smoothness, and starting and ending at baseline level, and Gaussian temporal autocorrelations; in the second case, a set of predefined prior HRF shapes, and many priors for modelling the noise distribution and autocorrelation.

The HRF extraction method described here is *data-driven *instead of model-driven. It is based on Fourier-wavelet regularised deconvolution, *ForWaRD *for short, developed originally for denoising and deblurring of images [17]. *ForWaRD *combines frequency-domain deconvolution for identifying overlapping signals, frequency-domain regularisation for suppressing noise, and wavelet-domain regularisation for separating signal and noise. It is related to recent wavelet-based deconvolution techniques [18-20], with the advantage that the roles of signal (for fMRI: sparse, high frequency) and response (for fMRI: smooth, low frequency) can be interchanged: unlike other wavelet- and vaguelette-based deconvolution methods, *ForWaRD *does the first step entirely in the frequency domain.

The method described in this paper uses an fMRI data set and the stimulus time pattern, and produces a time series of image volumes, which contains the HRF in each voxel. Compared to the simple extraction methods above, this method has the advantage of taking overlapping responses into account. On the other hand, it is much simpler than the Bayesian ones described above, in that it does not rely on shape assumptions/priors of the HRF: the extracted time points are determined only by the fMRI signal and the stimuli: the method is data-driven. This important property means that the extracted HRF is not biased by any a *priori *model. The only requirements for *ForWaRD *are (*i*) LTI responses and (*ii*) separability of signal and noise in the frequency/wavelet domain. Studies of the linearity of the BOLD response indicate that (*i*) is a reasonable assumption, and given the smoothness of the HRF and the availability of smooth wavelet basis functions, (*ii*) is satisfied as well. Another important advantage is the fact that wavelet-based signal processing methods are much better in preserving the 1/*f*-type temporal autocorrelations that are found in fMRI time signals than time-domain methods [21]. Models that do not preserve this structure yield biased results, leading to an increase in false positives [21,22]. Finally, our method is fast: HRF extraction from a single fMRI data set takes about the same time as spatial resampling.

To avoid possible misunderstandings we would like to stress that our method only concerns the relation between stimulus and haemodynamic response, but does not make any statement about the underlying neural response. Although it is known from the work by Logothetis et al. [23] that the BOLD response in the visual cortex of the monkey brain roughly corresponds to a convolution of the neuronal local field potential (LFP) with some neural response function (NRF), the exact form of this coupling is still unknown and remains an area of active research. In our method, we describe the haemodynamic response as a convolution of the *stimulus *(not the LFP) with an impulse response function, i.e., the HRF. Given the stimulus pattern our method can recover the HRF, but we do not claim to be able to extract the NRF via deconvolution.

The output time series can be used to determine subject-specific, group-specific, and region-specific HRFs, *e.g*., by averaging time signals in the corresponding regions. We demonstrate this in an fMRI experiment using two acquisitions, where the HRF extracted from the first fMRI time series is used to predict responses in a second experiment. A functional description for the HRF is found by fitting a combination of two gamma density functions with variable parameters for height, dilation, peak location and lag. We show that contrast and localisation improve by using the extracted HRF instead of the canonical HRF from the SPM2 program (a sum of two gamma density functions (GDFs) with fixed parameters).

The remainder of this paper is organised as follows. Section 2.2 reviews the *ForWaRD *method for regularised deconvolution. Sect. 2.2.4 describes how *ForWaRD *is used for HRF extraction, and it describes how the method was tested on simulated noisy time series and demonstrates a possible application using event-related fMRI data. Sect. 4 contains some general conclusions.

## 2 Methods

This section briefly reviews the standard General Linear Model (GLM) in fMRI, and describes the *ForWaRD *method [17] for extracting the HRF.

### 2.1 The General Linear Model

Statistical parametric mapping (SPM) is a common analysis method for fMRI. It (*a*) computes a statistic for every voxel using the GLM, (*b*) chooses a threshold based on the parameters of the noise and multiple testing correction; (*c*) thresholds the statistic map.

The GLM describes the response in an fMRI experiment as a weighted sum of *explanatory signals*. An explanatory signal is the response of one stimulus type. Let the matrix ***Y***_[*T*×*N*] _denote the fMRI data set, whose elements ***y***_*ij *_have time index *i *= 1, ..., *T *and position index *j *= 1, ..., *N *. In the GLM,

(1) ***Y ***= ***X***_*β *_+ ***e***.

***X***_[*T*×*M*] _is the *design matrix*, whose columns are the explanatory signals, and are multiplied by the weights in matrix ***β***_[*M*×*N*]_. Matrix *e*_[*T*×*N*] _is the residual signal for each ***y***_*ij*_. A least-squares estimate ***b ***for ***β ***is given by (***X***^*T*^***X***)^-1^***X***^*T*^***Y ***. Assuming a Gaussian temporal distribution of the residuals (this follows from the GLM if the temporal noise in ***Y ***is Gaussian), standard hypothesis testing can be used to assess the significance of the elements of ***b***.

### 2.2 Deconvolution, ForWaRD, and HRF extraction

In the GLM, a single response *g *to a pattern *f *of stimuli of the same type, is a convolution of *f *with the impulse response *h*, plus an additive term representing noise and other confounding effects.

#### 2.2.1 Deconvolution

Discrete circular convolution, denoted by '*', corresponds to multiplication in the frequency domain:

(2) *g*(*n*) = (h * *f*)(*n*) + *e*(*n*)     *G*(*k*) = *H*(*k*)*F*(*k*) + *E*(*k*),

capital letters denoting Fourier transforms of the corresponding lower-case signals. In the absence of noise, and given *g *and *f*, it is possible to compute an estimate $\tilde{h}$ of *h *through deconvolution. In the frequency domain, the Fourier transform $\tilde{H}$ of $\tilde{h}$ is obtained by pointwise division:

(3) $$\begin{array}{cc} \tilde{H}(k)=\{\begin{array}{ll} H(k)+\frac{E(k)}{F(k)}, & \text{if}|F(k)|>0\\ 0, & \text{otherwise}, \end{array} & k=1...N. \end{array}$$

**Figure 1:**
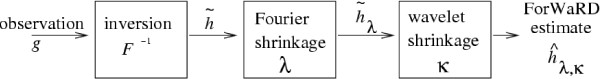
*ForWaRD *HRF extraction scheme. Fourier shrinkage (determined by *λ*) is applied to partially attenuate noise amplified during the inversion step. Subsequent wavelet shrinkage (determined by *κ*) effectively attenuates the residual noise.

**Figure 2:**
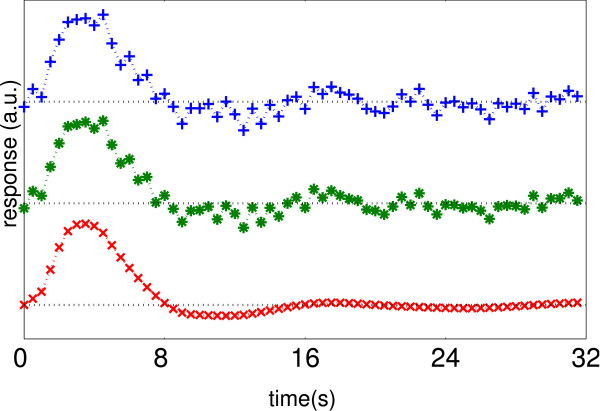
Stages of *ForWaRD*. HRF coeffcients after: frequency domain inversion (+); frequency domain shrinkage (*); wavelet domain Wiener shrinkage (×).

Deconvolution of a noisy signal is an ill-posed problem. A unique solution may not exist, be meaningless, or at best unstable: if noise is amplified at frequencies *k *where *F*(*k*) is close to zero, parts of the unmodelled signal ***e ***may appear in the extracted response (see Fig. 2i). An ill-posed problem can be *regularised *by adding extra information (or constraints) to the problem, so that its solution approximates the noise-free case [24]. Examples of such constraints are: minimising the norm between the solution and the data (minimum-norm), and making the solution more stable (smooth around optimum). *ForWaRD *uses regularisation methods in the frequency and wavelet domains (see Fig. 1) to overcome this problem.

#### 2.2.2 Fourier shrinkage

Frequency-domain shrinkage attenuates the noise after the pointwise division, by multiplying each frequency coeffcient $\tilde{H}$ (*k*) by a factor *λ *(*k*). Two popular methods are Wiener shrinkage and Tikhonov shrinkage [17]:

(4) $$\begin{array}{c} {\tilde{H}}_{\lambda }(k)=\tilde{H}(k)\lambda (k)\\ \lambda (k)=\{\begin{array}{ll} \frac{|F(k){|}^{2}}{|F(k){|}^{2}+\tau } & \left(\text{Tikhonov}\right)\\ \frac{|F(k){|}^{2}}{|F(k){|}^{2}+\alpha N\sigma_{\varepsilon }^{2}/|H(k){|}^{2}} & \left(\text{Wiener}\right) \end{array} \end{array}$$

Here $\sigma_{\varepsilon }^{2}$ is the variance of the noise *e*(*n*) in (2). The remarks at the end of this subsection explain how to estimate $\sigma_{\varepsilon }^{2}$ and determine optimal regularisation parameters *α *and *τ*. An estimate of the spectrum |*H*(*k*)|2 of the unknown response function needed for Wiener shrinkage is obtained by the iterative algorithm of Hillery and Chin [[25], Sect. 4]. The estimated response function ${\tilde{h}}_{\lambda }$ is the inverse Fourier transform of ${\tilde{H}}_{\lambda }$ (see Fig. 2ii).

#### 2.2.3 Wavelet shrinkage

Shrinkage in the wavelet domain is done using wavelet-domain Wiener shrinkage (WDWS), which reduces the noise and preserves details in the signal [17,26]. The discrete wavelet transform describes a sampled signal *c*^0 ^of length *N *as a sum of localised basis functions. A discrete wavelet transform with *J *levels of decomposition J∈ℕ recursively splits the signal into an approximation part *c*_*J *_and detail signals *d*_1_, *d*_2_, ..., *d*_*J*_, which are weighted sums of shifted and dilated versions of a *scaling function φ *and an associated *wavelet ψ*, respectively. The fast wavelet transform [27], which performs downsampling at each level, is not shift-invariant. *ForWaRD *uses a shift-invariant discrete wavelet transform (SI-DWT), which uses a polyphase decomposition (subsampling for all shifts) [28]. Reconstruction is possible via the inverse transform, denoted by SI-IDWT.

WDWS is performed via two wavelet transforms (for details, see [[17], section IV.C]). First, a wavelet transform of ${\tilde{h}}_{\lambda }$ is computed using (*φ*_1_, *ψ*_1_). A pilot estimate is obtained via scale-dependent thresholding of the detail coeffcients ${\left\{d_{1}^{j}(n)\right\}}_{1}^{J}$, resulting in thresholded detail coeffcients ${\left\{{\bar{d}}_{1}^{j}(n)\right\}}_{1}^{J}$, *n *=1,...,*N*. Another wavelet transform of ${\tilde{h}}_{\lambda }$ with wavelet basis (*φ*_2_, *ψ*_2_) yields detail coeffcients ${\left\{d_{2}^{j}(n)\right\}}_{1}^{J}$. These are shrunk by wavelet-Wiener filtering coeffcients:

(5) $$\begin{array}{cc} {\hat{d}}_{2}^{j}(n)=d_{2}^{j}(n)\kappa_{j,n} & \kappa_{j,n}=\frac{|{\bar{d}}_{1}^{j}(n){|}^{2}}{|{\bar{d}}_{1}^{j}(n){|}^{2}+\sigma_{j}^{2}} \end{array}$$

Here $\sigma_{j}^{2}$ is the noise variance at level *j*. Finally, the *ForWaRD *estimate ${\tilde{h}}_{\lambda ,\kappa }$ is the SI-IDWT of the shrunk coeffcients $\left(c_{2}^{J},{\left\{{\hat{d}}_{2}^{j}\right\}}_{j=1}^{J}\right)$ using wavelet basis (*φ*_2_, *ψ*_2_), see Fig. 2iii.

Remarks:

• The variance *σ*_*e *_is estimated based on the median absolute detail (MAD) coeffcient of a one-level wavelet transform [[29], §5.4] of the the noise *e*(*n*).

• In the frequency-domain shrinkage step, the parameters *τ *(Tikhonov) and *α *(Wiener) can be tuned to minimise the mean squared error (MSE) between the estimate ${\tilde{h}}_{\lambda ,\kappa }$ and the exact response function *h*. The exact MSE cannot be computed, but it can be approximated very accurately, see [[17], section VII].

• A complete Matlab implementation of the original *ForWaRD *algorithm, on which our method is based, is available ^1^

#### 2.2.4 Using *ForWaRD *for HRF extraction

The *ForWaRD*-based HRF extraction scheme (see Algorithm 1) works as follows: The BOLD response to a stimulus pattern *f *is relative to the baseline. Low-frequency trends in the baseline may contaminate the extracted HRF. Trends are removed beforehand by a standard wavelet-based technique [30]: transform each time signal (with length *N*) to the wavelet domain, using a fast wavelet transform (FWT) of log_2_(*N*) - 3 levels; remove the detail coeffcients, and subtract the low-scale signal from the time series. The influence of the detrending on the *ForWaRD *estimates is minimal, because the low-pass trends are in a much lower frequency band than the HRF. Processing takes place on a voxel-by-voxel basis, so images can be partitioned to reduce the computation load. The output is a time series of volumes which, inside activated brain areas, contain the HRF. Implementation has been performed in Matlab (The Mathworks, USA).

**Algorithm 1 ***ForWaRD*-based HRF extraction scheme.

1:   **for all **Voxels **do**

2:      load the discrete time series *g *and stimulus pattern *f*;

3:      compute and subtract the time series mean;

4:      remove low-frequency trends;

5:      apply *ForWaRD *to the mean-corrected and detrended signal *g *and stimulus pattern *f *to obtain the estimate ${\tilde{h}}_{\lambda ,\kappa }$ of the HRF coeffcients.

6:   **end for**

### 2.3 Tests on simulated fMRI time signals

**Figure 3:**
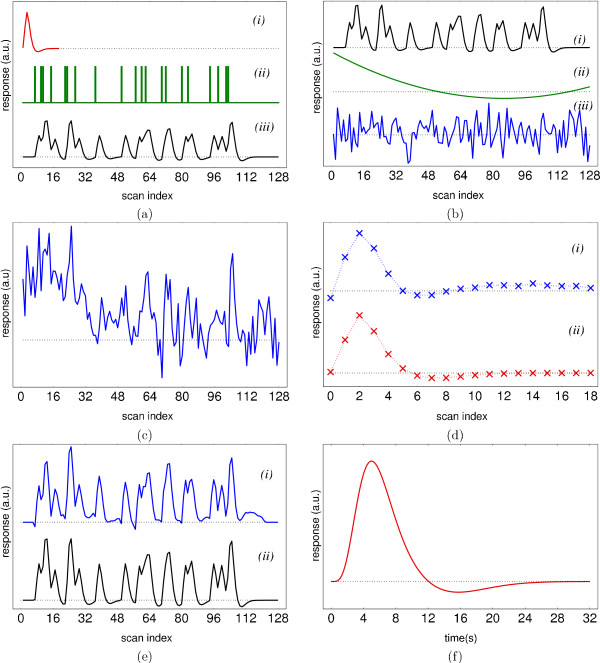
Test set-up. (a) The fMRI response as an LTI signal: HRF (*i*), stimulus pattern (*ii*), and the activation signal (*iii*) as (*i*) convolved with (*ii*). (b) In the GLM, confounds such as trends (*ii*) and noise (*iii*) are added to the response (*i*). (c) The total response: activation signal + confounds + noise. (d) The *ForWaRD*-reconstructed HRF (*i*) compared to the original (*ii*). (e) The activation signal using the extracted (*i*) and original (*ii*) HRF. (f) The *canonical HRF*, HRF_spm_, see Eq. (6).

The routine presented in Sect. 2.2.4 was tested on signals with varying properties (SNR, temporal resolution, low-frequency trends) with different settings of the routine itself (decomposition level, wavelet filters, etc.). Figures 3a–d shows the test setup: (a) convolve a stimulus pattern with a known HRF to obtain the activation signal; (b) add noise and a low-pass trend to it to obtain the total response; (c) recover the HRF from this total response, and (d) reconstruct the activation signal by convolving the stimulus pattern with the recovered HRF. The MSE between the activation signal and the reconstructed version was used to measure the reconstruction accuracy.

#### 2.3.1 Simulation of fMRI time signals

**Figure 4:**
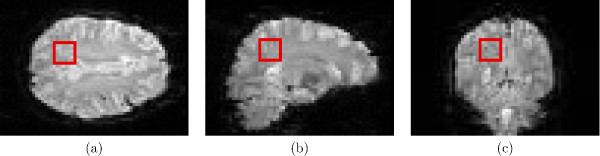
Area sampled from EPI data: (a) transverse, (b) sagittal, (c) coronal.

128 EPI scans of a subject at rest were acquired on a 3T Intera system (Philips Medical Systems, The Netherlands), with repetition time (TR) 3 s, image size 64 × 64 × 46 voxels of 3.5 × 3.5 × 3.5 mm^3^. A total of 512 noise time signals were collected from a region of 8 × 8 × 8 voxels (see Fig. 4). A randomised stimulus pattern *f*(*n*), *n *= 1, ..., *N *was obtained by thresholding a vector of random values. The stimulus *f*(*n*) *n *= 1, ..., *N *was convolved with an impulse response function *h*(*n*), in this case the canonical haemodynamic response function HRF_spm_, to describe the activation signal *s*(*n*). HRF_spm_(*t*) (see Fig. 3f) is the difference of two gamma density functions (GDF),

(6) $${\text{HRF}}_{\text{spm}}(t)=\gamma_{6,1}(t)-\frac{1}{6}\gamma_{16,1}(t),$$

where the GDF *γ*_*m*, *l*_(*t*) has the form:

(7) $$\begin{array}{cc} \gamma_{m,l}(t)=\frac{l^{m}t^{m-1}e^{-lt}}{\Gamma (m)}, & \Gamma (m)=\int_{0}^{\infty }t^{m-1}e^{-t}\text{d}t. \end{array}$$

**Figure 5:**
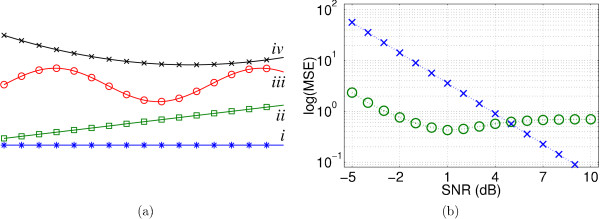
(a) Low-frequency trends: (*i*) flat, (*ii*) linear, (*iii*) sinusoidal, (*iv*) quadratic. (b) log_10_(MSE) of noisy (×) and reconstructed (o) signals given the input SNR.

Four types of low-frequency trends: flat, linear, sinusoidal and quadratic (see Fig. 5a) were added to the signal. The SNR of the time signals was set by choosing the standard deviations *σ*_*s *_of the signal and *σ*_*e *_of the noise, as well as the scalar *m*_*n *_such that

(8) $$\text{SNR}=10\log_{10}\frac{\sigma_{s}}{m_{n}\sigma_{e}}.$$

Trends with standard deviation *σ*_*t *_> 0 were scaled by *m*_*t *_so that *m*_*t*_*σ*_*t *_= *m*_*n*_*σ*_*e*_. The time signal in each voxel was the sum {EPI noise} + {activation} + {trend} (Fig. 3b). Tests included time signals with varying (*a*) input SNR, (*b*) low-frequency trends, (*c*) repetition time (TR), and (*d*) response onset.

#### 2.3.2 Reconstruction of activation signals

The HRF was extracted from each time signal by the *ForWaRD*-based routine, and the mean HRF was used to reconstruct the activation signal by convolving it with the stimulus pattern. The following settings of the *ForWaRD*-routine were varied: (*a*) type of frequency shrinkage, (*b*) levels of the wavelet transform, (*c*) wavelet-domain threshold level, and (*d*) the wavelet basis. The default values were: SNR 0 dB, no trend, TR 2 s, onset delay 0 s, Tikhonov shrinkage, *τ *0.1, decomposition level 3, *θ *3, *φ*_1 _Daubechies-4, *φ*_2 _Daubechies-3 [31].

### 2.4 Event-Related fMRI experiments

An application of the HRF extraction method is demonstrated in an event-related fMRI experiment. HRF coeffcients were extracted from the fMRI data set, and HRFs were computed by fitting a model function to HRF coeffcients (both whole-brain and region-of-interest). These HRFs were then used in a subsequent GLM-based analysis.

#### 2.4.1 Fixed-ISI experiment

The subject in the MRI scanner had to make a fist on the appearance of a visual stimulus, and relax after 1 s. Cues were given on a white screen placed inside the scanner: a red disc was a cue to make a fist, a white disc meant that the subject had to rest. The experiment consisted of 156 scans, acquired as described in Sect. 3.1. Cues were given every 24 s (8 scans × 3 s, no jittering), starting at scan 2. Increased task-related activity was expected in the motor cortex, the premotor cortex, the supplementary motor area and the cerebellum.

The EPI data were denoised with a wavelet-based technique [32], using SUREShrink in the wavelet domain [29]. Realignment, normalisation, and statistical analysis were done with SPM2 [2]. Slice timing correction was not applied. The design matrix ***X ***contained a set of 6 Fourier basis functions (3 sines, 3 cosines) modulated by a Hanning window, in the time interval of 8 scans after each stimulus, and a constant signal modelling the time series mean. The Fourier basis was used to model a large class of signals using only few assumptions.

The variance ratio [33] was computed in each voxel, and an *F*-test was used to determine significance. False discovery rate (FDR) control [34] with *q *= 0.05 was used to correct for multiple hypothesis testing.

#### 2.4.2 Random-ISI experiment

A second experiment used a random stimulus sequence, created by thresholding a sequence of random values (39 stimuli, ISI mean: 6.05 scans, *σ *: 4.31 scans, no jittering). The number of scans was 256, scanning parameters and preprocessing were as in Sect. 3.1.

Due to overlapping responses, the windowed Fourier basis set could not be used. It was replaced by the canonical HRF from the SPM2 program and its time and dilation derivatives. An F-test of the variance ratio with FDR *q *= 0.05 was used to assess significance.

#### 2.4.3 Extracting and modelling HRFs from the time series

A stricter FDR-corrected threshold (*q *= 0.0001) was applied to the variance ratio maps, sampling the HRF in a smaller number of voxels.

A continuous HRF was obtained by fitting a generalised version of HRF_spm _(6) to the coeffcients returned by *ForWaRD *and selective averaging. We use HRF_gam _to denote the difference of two GDFs with 8 parameters, *i.e*., *H*(eight), *D*(ilation), *P*(eak location) and *L*(ag) of both GDFs:

(9) $${\text{HRF}}_{\text{gam}}(t)=H_{1}\gamma_{P_{1},D_{1}}(t-L_{1})-H_{2}\gamma_{P_{2},D_{2}}(t-L_{2}).$$

Levenberg-Marquardt's nonlinear curve-fitting algorithm was used to determine these parameters and 95% prediction intervals for the fitted functions.

#### 2.4.4 Using fitted HRFs to model responses

HRFs measured from an fMRI data set cannot be used to test for activation in that same data set: a model must be specified *a priori*, so that inferences are not made from models that are determined by the data itself. We tested for activation in the random-ISI experiment with the HRFs fitted to the coeffcients extracted from the fixed-ISI data, and *vice versa*. The GLM was estimated using the stimulus times and the fitted HRFs, and their correlation the fMRI time signals was computed. Significance was determined via a one-sample *t*-test, and FDR control with *q *= 0.05 was used to correct for multiple hypothesis testing.

## 3 Results

### 3.1 Simulated fMRI time signals

The properties of the signal and parameters of the extraction routine that noticeably influenced the original activation signal *s*(*n*) and its reconstruction *r*(*n*), are listed below.

#### 3.1.1 Output MSE as a function of input SNR

Figure 5b shows the MSE for various input SNR values. For input SNRs up to 9 dB the MSE decreases; it increases above 9 dB.

#### 3.1.2 Choice of shrinkage type and *τ *parameter

**Figure 6:**
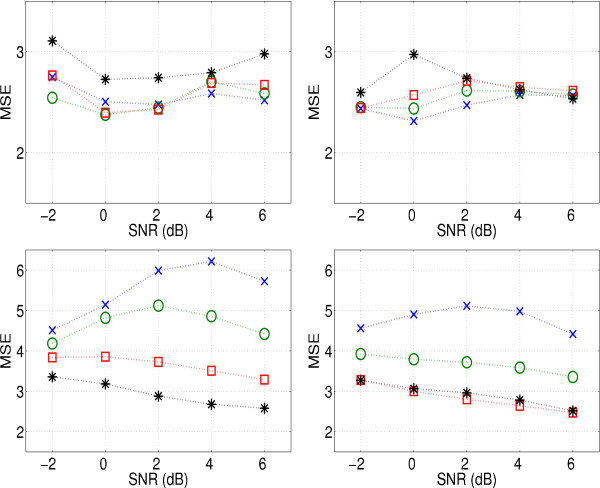
Output MSEs of Tikhonov (left) and Wiener (right) shrinkage with a TR of 0.5 s (top) and 3 s (bottom), for different input SNRs and a varying regularisation parameter *τ *× *τ *= 0.01, o *τ *= 0.1, □: *τ *= 1, *: *τ *= 10.

Wiener shrinkage uses an iterative algorithm [25] to estimate |*H*|^2 ^(see (4)), the number of iterations was limited to 10. Figure 6 shows the MSE for both types of frequency domain shrinkage, varying SNR and regularisation parameter *τ*. For low SNR and heavy regularisation (*τ *≥ 1), Tikhonov regularisation outperforms Wiener shrinkage. For higher SNRs or mild regularisation, Wiener shrinkage performs better. The best value for *τ *depends on the shrinkage type, the SNR and the TR. For short TR, mild regularisation (*τ *≤ 0.1) is preferable. A long TR requires heavy regularisation.

#### 3.1.3 Different response delays

**Figure 7:**
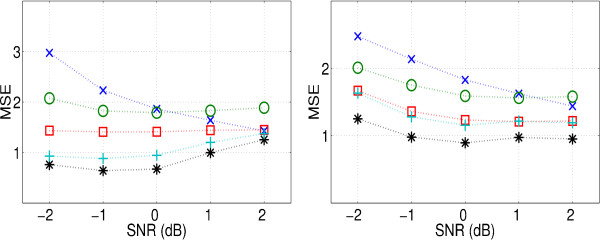
Output MSE for varying response onset delays, for Tikhonov (left) and Wiener (right) shrinkage, SNR = -2 dB (×), 0 dB (o), 2 dB (□), 4 dB (*), and 6 dB (+).

Figure 7 shows that HRFs with different onset delays can equally well be extracted with *ForWaRD*. The MSE hardly changes with different delays, indicating that the shape of the response is preserved. The increased MSE for negative shifts is caused by the fact that the HRF was only sampled post-stimulus.

#### 3.1.4 Different wavelet filters

We tested 15 different wavelet filters for (*φ*_1_, *ψ*_1_), as well as for (*φ*_2_, *ψ*_2_): Daubechies wavelets 1 ... 5 (the filter number indicates the number of vanishing moments), Daubechies' symmetric wavelets 2 ... 6 [35] (filter 1 corresponds to the Daubechies-1 filter), and Coiflets 1 ... 5 [35]. Different filters did not yield large differences in performance.

#### 3.1.5 Decomposition level and noise threshold

**Figure 8:**
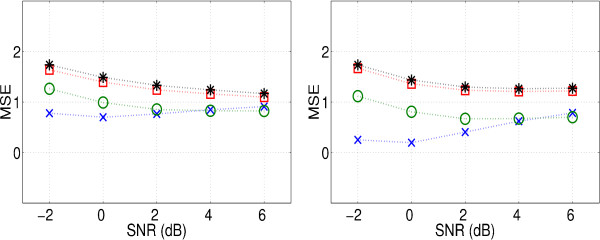
Output MSE for different SNRs, with various levels of decomposition and threshold levels. Left: *θ *= 2, right: *θ *= 3, with a two-level transform (×), three-level(o), four-level (□), five-level(*).

Figure 8 shows the MSE for different SNRs, different *θ *and different decomposition levels. We find that two-level decompositions produce the smallest errors for the lower SNRs, and three-level decompositions perform best for the higher. Four-level and five-level decompositions yield higher errors. A higher *θ *often yields a lower MSE.

#### 3.1.6 Different low-frequency trends

**Figure 9:**
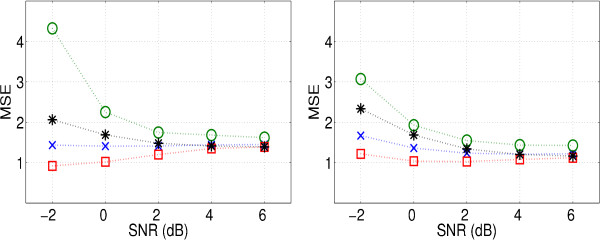
Output MSE for different SNRs and low-pass trends in the data. Left: Tikhonov shrinkage, right: Wiener shrinkage. No trend (×), a linear trend (o), a sinusoidal trend (□), or a quadratic trend (*).

Tests with low-frequency trends (see Fig. 5a) show that the type of frequency domain shrinkage changes the result. (see Fig. 9). The MSE is higher with Tikhonov than with Wiener shrinkage, especially for lower SNRs. Trends are not removed perfectly with either shrinkage type, but the extra information about *f *and the power spectral density of *h *in Wiener shrinkage make it less sensitive to trend residuals.

#### 3.1.7 Summary

Even though the default parameters (see Sect. 2.3.2) make the algorithm robust for a wide range of signals (different SNR, sampling frequency, etc.), the method is also robust changing its parameters, such as the wavelet filters and decomposition level. The MSE of the reconstructed signal was lower than the input MSE in most of the tested situations.

The robustness to onset delay makes *ForWaRD *an attractive alternative to other delay correction methods such as including a temporal derivative of the standard HRF in the model [16]: these only correct for small synchronisation errors, and are not usable when the HRF is not well modelled by the standard function.

### 3.2 Event-related fMRI: fixed-ISI

**Figure 10:**
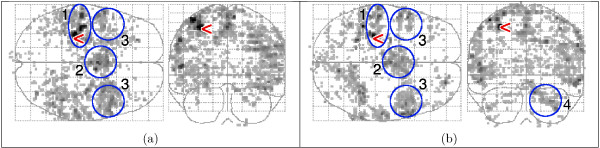
Maps of the variance ratio using the F-test with FDR *q *= 0.05. The highest value is indicated with a '<' sign. (a) Fixed-ISI experiment, range 3.86–20.63 and (b) random-ISI experiment, range 5.43–41.63 (b). The indicated areas are: l.motor cortex (1), supplementary motor area (2), premotor area (3), r.cerebellum (4).

**Figure 11:**
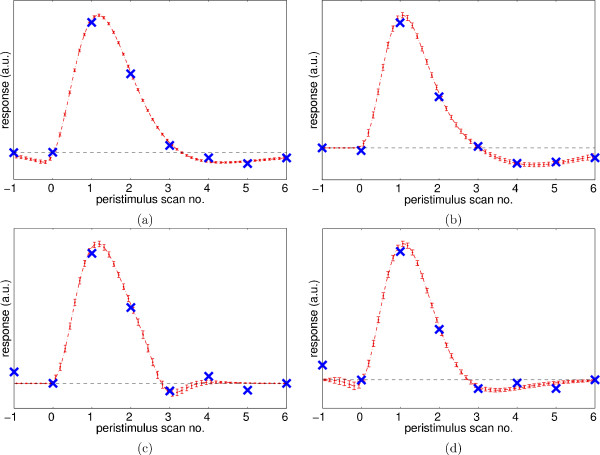
HRFs extracted from the fixed-ISI data by selective averaging (top row) and *ForWaRD *(bottom row). Left: whole-volume, right: region-specific. The extracted coeffcient are the × at each TR. Dotted lines: fits of HRF_gam _to the coeffcients. Error bars show the 95% confidence intervals for the fitted function.

Activation maps are shown in Fig. 10a as 'glass brain' maximum intensity projections (MIP) in two orthogonal directions. Low statistic values are shown in grey, high values in black. The highest value is indicated with a '<' sign. Activation was found in the expected areas, predominantly in the motor cortex. A whole-volume HRF was extracted from the post-stimulus volumes using selective averaging [9], by taking the mean response of each volume, weighted by the map of significant statistic values. A region-specific HRF in the 7 × 7 × 7-voxel neighbourhood of a selected voxel (see the '<' in Fig. 10a) was computed by using only the time signals from that region. Figure 11a–b shows the extracted HRFs.

The *ForWaRD *algorithm used 128 scans of the experiment, starting with scan 2 (first stimulus). Whole-volume HRF and region-specific HRF time points were computed using the post-stimulus time series (see Fig. 11c–d). The HRFs extracted by *ForWaRD *are similar to the results from selective averaging, except that the baseline of the *ForWaRD*-extracted HRF decreases. This is because the HRF does not return to baseline within the sampled interval (24 s), so in the GLM the response decreases at the end of every stimulus. In some cases (see Fig. 11c–d) this was fixed manually by setting the baseline to the last HRF coeffcient.

### 3.3 Event-related fMRI: random-ISI

The contrast of the random-ISI experiment was generally weaker than in the fixed-ISI experiment, but localisation was better, see Fig. 10b.

A post-stimulus time series was made with *ForWaRD*, using all 256 scans of the experiment. Selective averaging could not be used because of overlapping responses. With a random ISI, a much longer post-stimulus interval can be sampled [11]. The post-stimulus volumes produced by the extraction routine were used to create a whole-volume HRF and a region-specific HRF in the same way as in the fixed-ISI case.

**Figure 12:**
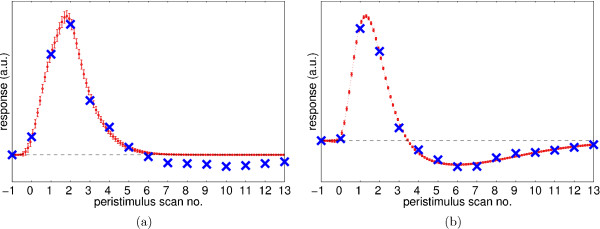
HRFs extracted from the random-ISI experiment by *ForWaRD*: whole-volume (a) and region-specific (b). ×: extracted HRF coeffcients. Dashed lines: function HRF_gam _fitted to ×. 95% prediction intervals for the fitted functions are shown as error bars.

Figure 12 shows the extracted HRF coeffcients and the fitted HRFs. A comparison between Fig. 11c–d and Figure 12 shows that the *ForWaRD*-extracted HRF coeffcients from the random-ISI design agree better with the model function than those from the fixed-ISI design, especially in the region-specific case. A possible explanation is that the fixed-ISI stimulus signal does not contain enough frequency information to do the Fourier inversion, resulting in a lower-quality estimate. In contrast, selective averaging does not work with overlapping responses and random ISIs.

### 3.4 Activation detected with fitted HRFs

**Figure 13:**
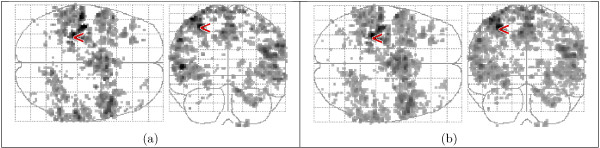
Activation in the fixed-ISI data set, using the *F*-test with FDR q = 0.05. The highest value is indicated with a '<' sign. HRFs extracted from the random-ISI data set by *ForWaRD *and modelled with HRF_gam_. (a) whole-volume HRF, range 3.08–10.62. (b) region-specific HRF, range 2.99–12.72.

**Figure 14:**
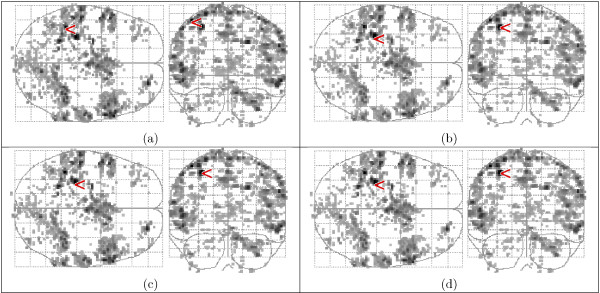
Activation maps of the random-ISI data set, using the *F*-test with FDR *q *= 0.05. The highest value is indicated with a '<' sign. HRFs modelled by HRF_gam_, coeffcients extracted from the fixed-ISI data set by selective averaging with whole-volume HRF (a, range 3.10–10.11) and region-specific HRF (b, range 3.10–10.21), and *ForWaRD *with whole-volume HRF (c, range 3.11–10.15) and region-specific HRF (d, range 3.13–10.10).

Figure 13 shows the activation detected in the fixed-ISI dataset with the HRFs extracted by *ForWaRD *from the random-ISI dataset. There is good correspondence between the maps of the whole-volume (a) and region-specific (b) HRFs, respectively, and the detected activations match the expected pattern (see Fig. 10). Figure 14 shows the activation detected in the random-ISI dataset with the HRFs from the fixed-ISI dataset, using both selective averaging (a-b) and *ForWaRD *(c-d), which are in very good agreement. Where selective averaging is possible, *ForWaRD *yields results very similar to those produced by selective averaging. The statistical maps for *ForWaRD *between the fixed-ISI and random-ISI time series are in good correspondence.

**Figure 15:**
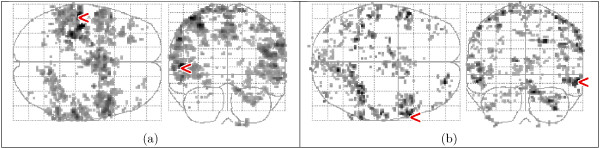
Activation maps of the fixed-ISI data set (a, range 3.10–10.80), and the random-ISI data set (b, range 3.32–8.61), using HRF_spm_. The highest value is indicated with a '<' sign.

**Figure 16:**
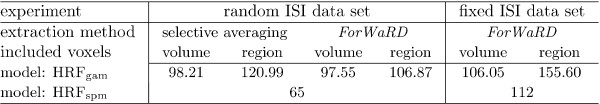
Maximum variance ratio values found in the tests.

An analysis was also performed performed with the general HRF_spm_. The activation maps in Fig. 13 are in very good correspondence with Fig. 15a, indicating that both HRF_spm _and HRF_gam _model the data well. The values for the variance ratio in Fig. 16 for the fixed-ISI experiment show that all models explain the data well, and HRF_gam _with the regional HRF yields the best fit to the data.

Figures 14 and 15b show that HRF_spm _yields a poor fit to the data; this is also shown in Fig. 16. The higher variance ratios for HRF_gam _indicate that a larger portion of the measured variance is explained by the model, and that the residual of the GLM (1) is small. The fitted region-specific HRFs generally perform better than whole-volume HRFs, and the maps of detected activation indicate that the fitted HRFs do not only detect activation in the region from which they were extracted, but that they are general enough to also detect activation in other areas, corresponding to the predicted activated regions (see the blue ellipses in Fig. 10).

## 4 Conclusion

We have developed a technique to extract HRF coeffcients from fMRI time series based on the *ForWaRD *deconvolution technique. Frequency-domain deconvolution allows extraction of the HRF even when the responses to subsequent stimuli overlap, and the sensitivity to noise of frequency-domain deconvolution is compensated by Wiener or Tikhonov shrinkage in the frequency domain, followed by wavelet-domain Wiener shrinkage. Before applying *ForWaRD*, low-frequency trends are removed from the time signal with a standard wavelet-based method. Tests of the extraction routine using simulated activation, several types of trend and noise from a real fMRI time series, demonstrate its robustness. Results show that the method is robust to trends in the data, and the performance does not differ much between the noise levels we tested. The output of our algorithm is a post-stimulus time series, representing the HRF coeffcients in every voxel. At present, the extraction method is capable of recovering one HRF from one time series. The algorithm used in this paper is entirely data-driven: it does not use a *priori *models of the data. Other advantages of this algorithm are its simplicity, *i.e*., the algorithm works independently of other pre- and postprocessing steps; and its speed and low computational complexity.

The default settings of the method used in the tests as well as in the fMRI experiments (see Sect. 2.3.2) lead to a good and robust performance of the extraction algorithm.

We have demonstrated the use of *ForWaRD*-extracted HRFs in combination with continuous HRF functions to predict event-related fMRI responses. Given the output of the extraction routine, continuous functions (in this case gamma densities) are fitted to the average HRF coeffcients in a region of a (statistical or anatomical) map.

Results from the experiments with extracted and fitted HRFs indicate that subject-specific and region-specific HRFs lead to stronger contrasts and better localisation than a standard HRF. The results with the random-ISI data suggest that it is possible to use *ForWaRD *in combination with relatively complex prior experiments to extract HRFs, and that it is beneficial to use these HRFs instead of standard functions for detection and modelling of subsequently acquired data. The advantage of using a single, tailored HRF over a model that spans several basis functions, is that the statistical analysis is more specific and more powerful, resulting in a stronger contrast and better localisation.

A possible extension of the method is the extraction of multiple HRFs from one or multiple experiments. Deconvolution in the frequency domain of multiple waveforms has already been done in ERP research [36]. The *ForWaRD*-based method may be extended in a similar way.

## Competing interests

The author(s) declare that they have no competing interests.

## Authors' contributions

AMW wrote the software, did the analyses and wrote most of the paper. JBTMR designed critical parts of the method and coauthored the paper. HH led the image acquisition.

## Note

Figure 16 Maximum variance ratio values found in the tests.

^1^

## Pre-publication history

The pre-publication history for this paper can be accessed here:


